# Transcriptomic characters of cochlear vascular cells with pericyte-driven angiogenetic activity

**DOI:** 10.21203/rs.3.rs-8535400/v1

**Published:** 2026-01-23

**Authors:** Pingting Wang, Yunpei Zhang, Zhiqiang Hou, Jinhui Zhang, Xiaorui Shi

**Affiliations:** Oregon Health & Science University; Oregon Health & Science University; Oregon Health & Science University; Oregon Health & Science University; Oregon Health & Science University

**Keywords:** Angiogenesis, Cochlea, Blood-labyrinth barrier (BLB), Endothelial cell, Pericytes, Single-cell RNA sequencing

## Abstract

The normal structure and function of inner-ear blood vessels, including the microvascular network of the stria vascularis (SV) within the blood-labyrinth barrier (BLB), are essential for auditory function. Despite this, the genetic and molecular characteristics of cochlear vasculature are largely unexplored. In this study, we used single-cell RNA sequencing to profile endothelial cells (ECs) and pericytes (PCs) from the adult mouse cochlea. We found a distinct genetic profile and a higher angiogenic potential than observed in the blood-brain barrier (BBB). Two subclasses of PCs were identified. Type 1 PCs, with high levels of α-smooth muscle actin (*Acta2*) and *Tagln*, are located on pre-/post-capillary zones. Type 2 PCs, characterized by low *Tagln* and high *Kcnj8/ Abcc9* levels, are found specifically in capillary regions. In an ex vivo explant model, both subclasses showed tip-like behavior during sprouting. Ligand-receptor analysis indicated active EC-PC communication. This communication is mediated by adhesive signals, gap junctions, and vesicle trafficking. Using dual fluorescent reporter mouse models, we showed for the first time that PCs can transition into tip cells by co-expressing NG2/PECAM-1 signals. This transition may occur from existing cells or progenitors within the vascular niche. Our findings define the molecular signature of cochlear vessels and identify PCs as targets to promote vascular regeneration. This could have implications for hearing restoration when cochlear blood flow is compromised.

## Introduction

The cochlea is a fluid-filled sensory organ that needs a tightly regulated microenvironment for auditory transduction [1–3]. This regulation is maintained by the blood-labyrinth barriers (BLB), which control solute movement between blood and inner-ear fluids (perilymph and endolymph) to maintain tissue homeostasis. A key BLB component is the interstitial fluid-blood barrier (ISFBB) of the stria vascularis (SV) [4, 5]. Prior work links ISFBB integrity to specialized endothelial tight junctions and low transcytosis, thereby restricting paracellular and vesicular transport and sustaining the endocochlear potential (EP) required for hair-cell function and hearing. Despite this functional importance, the molecular architecture of cochlear vasculature remains incompletely understood.

The ISFBB is supported by abundantly distributed strial PCs, specialized mural cells that ensheathe capillaries and share the basement membrane with ECs, allowing intricate cell-to-cell signaling between the two cell types [6]. Notably, the cochlea has the highest ratio of PCs to ECs among tissues [5]. PCs are essential for cochlear vascular integrity; loss of PCs leads to a leaky ISFBB [7]. However, how PCs communicate with ECs to maintain barrier function remains poorly understood. Previously, we showed that strial angiogenesis can be driven by PCs with tip-like behavior [8]. The EC-PC interactions that make this possible are still unclear. In this study, we isolated fresh ECs and PCs from adult mouse cochlea for single-cell RNA sequencing (scRNA-seq). We aimed to define EC transcriptional features, identify PC subclasses, and map EC-PC communication for the first time. We also examined ‘tip-associated’ transcripts in PCs and explored EC-PC dynamics using dual-reporter models and functional 3D explant assays. Cochlear vascular cells show a transcriptional bias toward angiogenesis-related pathways compared to the blood-brain barrier (BBB). This suggests there is greater remodeling competence in the adult cochlea [9].

PC ontogeny is classically attributed to embryonic mesenchyme and neural crest derivatives [10, 11], with tissue-specific contributions from mesodermal and ectodermal sources: for example, PCs in the brain, heart, and liver are derived from ectoderm, whereas PCs in most other organs typically originate from mesoderm [12]. Using inducible CreER reporters under the NG2 (Cspg4) promoter, a mural-cell marker [13], we performed lineage tracing and fate mapping to evaluate whether NG2-lineage progenitors contribute to adult angiogenesis in 3D explants. We identified NG2+ cells in the perivascular niche at E11.5-E13.5 that later acquired PC identity upon induction at E14.5-E18.5. In explant assays, both resident PCs and NG2-lineage progenitors contributed to sprouting, with PCs exhibiting PECAM-1 signal at the invasive front. Together, our study provides a transcriptomic and functional definition of strial vascular cells, highlights PC diversity and communication with ECs, and suggests that targeting PCs and their progenitors could restore vascular function and protect hearing in hypoxic or injury contexts, including noise exposure, aging, and Ménière’s disease.

## Results

### Transcriptomic profiling reveals homogeneous strial EC signatures with enrichment of angiogenesis-linked pathways

**Figure 1:**
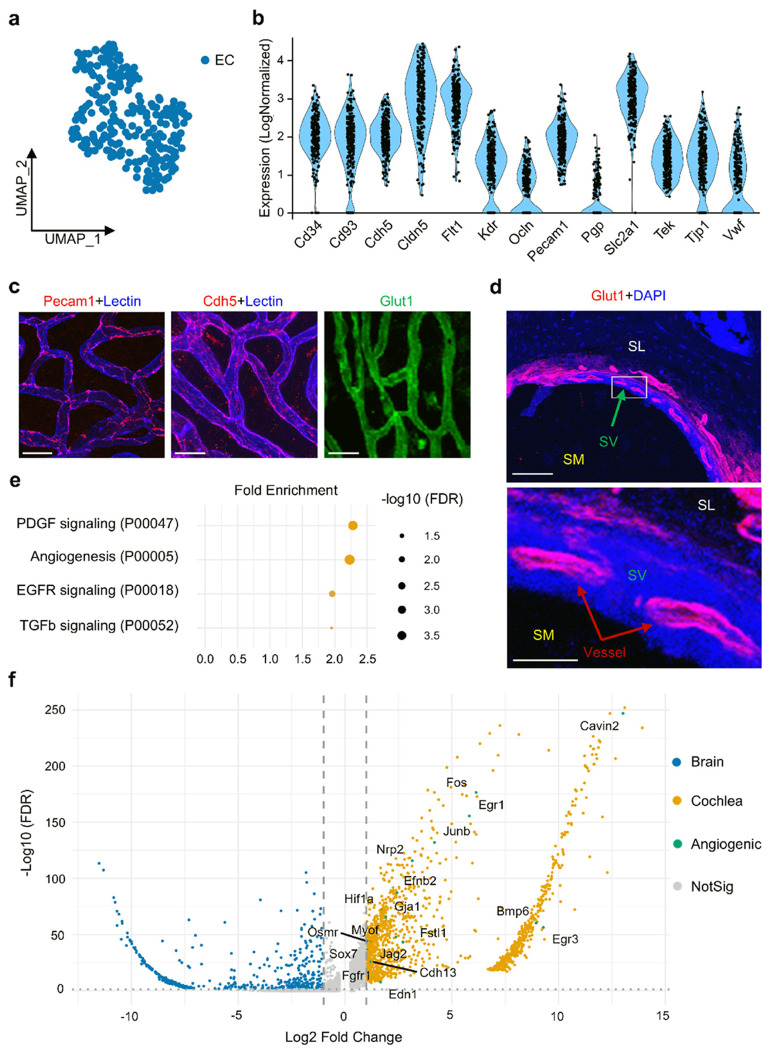
Transcriptomic and protein features of strial ECs (ECs). (a) UMAP showing a single, homogeneous EC cluster from adult mouse SV identified by Seurat (b) Expression of canonical EC markers: *Cd34, Cd93, Cldn5, Cdh5 (VE-cadherin), Flt1(Vegfr1), Kdr (Vegfr2), Olcn, Pecam1 (Cd31), Pgp (Abcb1), Slc2a1 (Glut1), Tek (Tie2), Tjp1 (ZO-1), Vwf*(c) Immunofluorescence staining of CD31, VE-Cadherin, and GLUT1 (SLC2A1) in SV; Scale bar: 20μm (d) Immunofluorescence staining of GLUT1 on cochlear cryosection. Top: low-magnification overview; bottom: high-magnification view of the boxed region; Scale bars: 50 μm (top), 10 μm (bottom);SM: Scala media, SL: Spiral ligament, SV: Stria vascularis (e) PANTHER pathway enrichment of genes upregulated in cochlear ECs vs. brain ECs (f) Volcano plot of differentially expressed genes: brain ECs (blue) and cochlear ECs (orange); angiogenesis-annotated genes up in cochlear ECs (green)

To identify EC features in the ISFBB, we performed scRNA-seq on freshly isolated ECs from 20 cochleae of Tie2-GFP endothelial fluorescence reporter mice. ECs were identified by distinguishing them from contaminating cell types based on Tie2 expression, with quality control (QC) processes to filter out low-quality cells and technical artifacts from the scRNA-seq data (Fig. S1a-d). UMAP projection of the dataset revealed distinct transcriptomic profiles specific to a single, uniform cluster of strial ECs (Fig. 1a), distinct from the pronounced heterogeneity observed in ECs from other organs, including the brain, lung, and kidney [14–16]. Although there appeared to be three clusters that were spatially mapped as adjacent and interdigitated within the stria (Fig. S1d), module scoring for arterial, venous, and capillary signatures failed to segregate them (Fig. S2a) [17]. Additionally, fixed tip/stalk separators that were not differentially stabilized were observed (Fig. S2b) [18], and our top-gene AUROC analysis did not identify any stable subtype-defining markers (Fig. S2c). Strial ECs expressed classic endothelial genes, including *Kdr* (*Vegfr2*), *Flt1* (*Vegfr1*), *Cldn5* (*claudin-5*), *Pecam1* (*Cd31*), *Cdh5* (*VE-Cadherin*), *Tek* (*Tie2*), and *Vwf* (Fig. 1b), with enriched adhesive protein expression of CD31, VE-Cadherin, and SLC2A1 (GLUT1) confirmed by immunofluorescence (Fig. 1c–d).

Further differential-expression (DE) analysis between cochlear ECs and brain ECs yielded a cochlear-upregulated gene set that, on PANTHER pathway analysis, was overrepresented in PDGF signaling (P00047), angiogenesis/blood vessel growth (P00005), EGF receptor signaling (P00018), and TGF-β signaling (P00052) (Fig. 1e) [9]. The volcano plot displays DE genes with brain ECs in blue and cochlear ECs in orange; angiogenesis-annotated genes up in cochlear ECs are highlighted in green (Fig. 1f). Together, our results demonstrate a specialized, transcriptionally homogeneous population of strial ECs with enhanced angiogenic potential in adult cochlea.

### scRNA sequencing identifies two subclasses of PCs located in distinct anatomical regions

**Figure 2:**
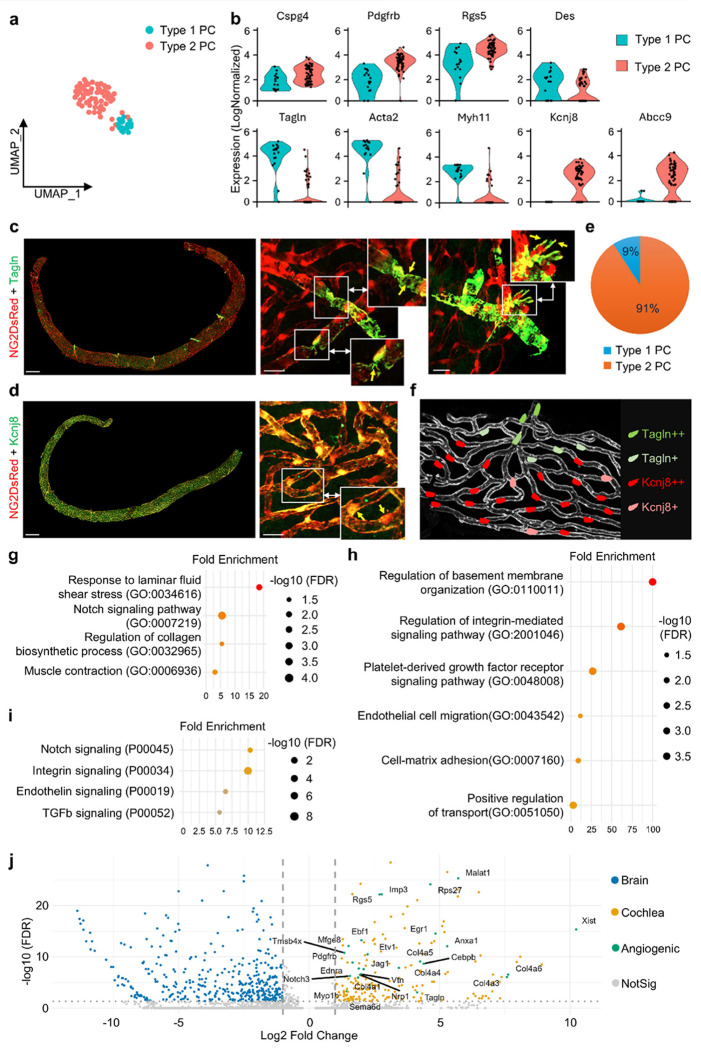
Heterogeneity of cochlear strial PCs revealed by scRNA-seq and validation. (a) UMAP of strial PCs showing two clusters (b) Violin plot of canonical PC markers (*Cspg4/ NG2, Pdgfrb, Rgs5, Des*) and subtype markers distinguishing Type 1 and Type 2 PCs (c)Immunofluorescence staining of TAGLN in SV, confirming spatial distribution of Type 1 PCs; Scale bars: left, 250 μm; right panels, 20 μm;Squared regions in the right panels are shown magnified on the right side;Yellow arrows in the magnified panels indicate characteristic PC processes encircling the vessel wall (d) Immunofluorescence staining of KCNJ8 in SV, confirming spatial distribution of Type 2 PCs; Scale bars: left, 250 μm; right, 20 μm;Squared region in the right panel is shown magnified on the right side; In the magnified panel, the left yellow arrow marks a KCNJ8++ PC and the right yellow arrow marks a KCNJ8+ PC (e) Quantification of Type 1 vs. Type 2 PC abundance (f) Schematic illustration represents spatial differences between Type 1 and Type 2 PCs (g) GO Biological Process enrichment (top 500 genes) in Type 1 PCs (h) GO Biological Process enrichment (top 500 genes) in Type 2 PCs (i) PANTHER pathway enrichment of genes upregulated in cochlear PCs vs. brain PCs (j) Volcano plot of differentially expressed genes: brain PCs (blue) and cochlear PCs (orange); angiogenesis-annotated genes up in cochlear PCs (green)

The cochlear SV contains a rich PC population that plays a crucial role in maintaining cochlear vascular stability and integrity [19]. To investigate their transcriptional features in the SV, we isolated PCs from the cochleae of 10 NG2-DsRed PC reporter mice, for a total of 20 cochleae. PCs were identified, distinguishing them from contaminating cell types based on NG2 expression, with quality control (QC) processes to filter out low-quality cells and technical artifacts from the scRNA-seq data (Fig. S3a-d). UMAP projection revealed two transcriptionally distinct PC clusters, in contrast to the relatively homogeneous strial ECs (Fig. 2a). Both clusters expressed canonical PC markers, including *Cspg4* (*NG2*), *Pdgfrb, Rgs5*, and *Des*, but could be further distinguished by the expression of subtype-specific genes (Fig. 2b). The first cluster, designated Type 1 PCs, was enriched for *Tagln* (which encodes transgelin), *Acta2* (α-smooth muscle actin), and other cytoskeletal regulators such as *Myh11* (Fig. 2b). These markers suggested a more contractile phenotype consistent with mural cell function [20, 21]. Immunofluorescence confirmed the presence of TAGLN+ PCs in pre- and post-capillary regions of the SV, where they co-localized with NG2+ mural structures; zoomed panels highlighted characteristic cell-leg processes encircling vessels (Fig. 2c). The second cluster, designated Type 2 PCs, exhibited low expression of *Tagln* and was localized across true capillary networks, along with prominent expression of *Kcnj8* and *Abcc9*, in addition to other PC hallmarks (Fig. 2b, 2d). Quantification of subtype abundance was shown in Fig. 2e, and a schematic summarized their spatial relationship (Fig. 2f). Gene Ontology (GO Biological Process) analysis of top 500 genes indicated that Type 1 PCs were enriched for response to laminar fluid shear stress (GO:0034616), muscle contraction (GO:0007219), Notch signaling pathway (GO:0007219), and regulation of collagen biosynthetic process (GO:0007219) (Fig. 2g). Type 2 PCs were enriched for regulation of basement membrane organization (GO:0110011), PDGF receptor signaling pathway (GO:0048008), positive regulation of integrin-mediated signaling pathway (GO:2001046), cell-matrix adhesion (GO:0007160), EC migration (GO:0043542), and positive regulation of transport (GO:0051050) (Fig. 2h).

To further define strial PCs relative to other tissue contexts, we performed differential expression (DE) analysis of cochlear PCs versus brain PCs. PANTHER pathway analysis of cochlea-upregulated genes showed overrepresentation of angiogenesis-linked programs, including Notch signaling (P00045), Integrin signaling (P00034), Endothelin signaling (P00019), and TGFb signaling (P00052) (Fig. 2i) [9]. The volcano plot displayed DE genes (brain PCs, blue; cochlear PCs, orange), with angiogenesis-annotated transcripts up in cochlear PCs highlighted in green (Fig. 2j), including the *Col4a* series, *Vtn, Nrp1, Notch3*, and *Jag1*. Additionally, the expression of *Pdgfrb* and *Tagln* was higher in cochlear PCs than in brain PCs, reinforcing the identity and contractile properties of the Type 1 subset in the SV (Fig. 2j). Collectively, these findings demonstrated that cochlear PCs were not a homogeneous population but instead comprised two subclasses with discrete molecular signatures and spatial localization. This heterogeneity may underline the ability of PCs to both stabilize vascular architecture and contribute directly to angiogenesis in the cochlea.

### Transcriptional analysis reveals EC-PC active communication

**Figure 3:**
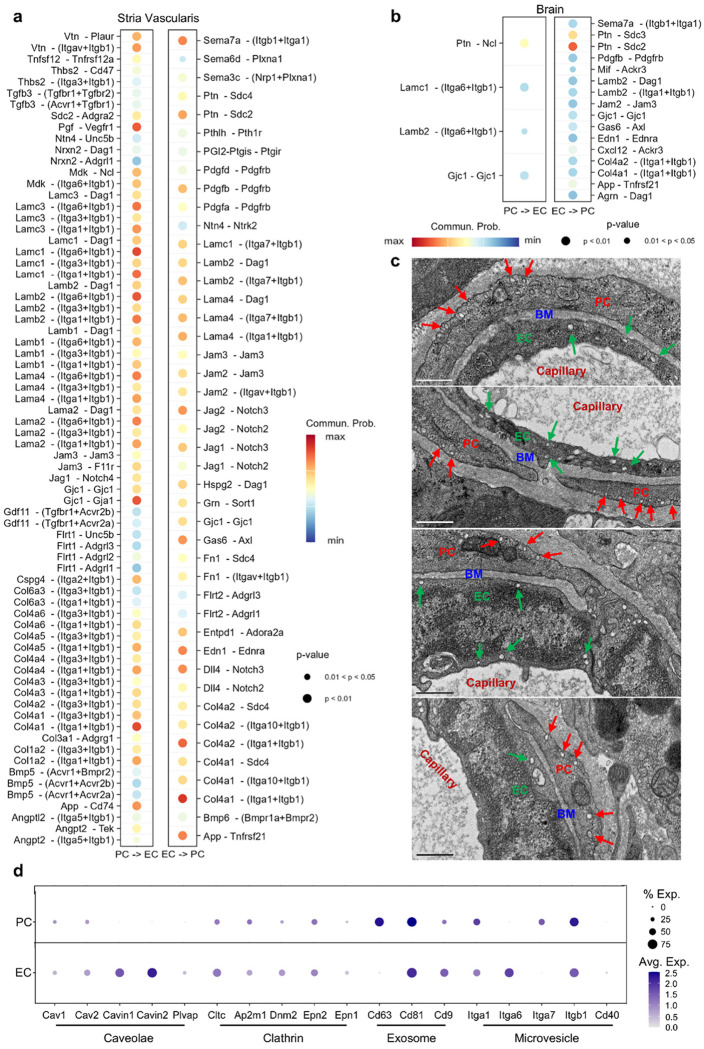
EC-PC communication and vesicle/trafficking programs in the stria vascularis. (a) Bubble plot (CellChat) of predicted ligand-receptor interactions between strial ECs and PCs;Dot size indicates p-value; color reflects communication probability (b) Bubble plot of predicted EC-PC interactions in the brain (c) Transmission electron microscopy (TEM) of SV ultrastructure, highlighting PCs (red), basement membrane (BM, blue), ECs (green), and capillary lumen (dark red). Colored arrows indicate representative vesicles in PCs and ECs;Scale bar: 500 nm (d) Vesicle/trafficking gene programs in strial PCs and ECs;Groups: caveolae core (*Cav1, Cav2, Cavin1, Cavin2, Plvap*), clathrin endocytosis (*Cltc, Ap2m1, Dnm2, Epn1/Epn2*), small-EV markers (*Cd63, Cd81, Cd9*), and microvesicle-associated integrins (*Itga1/Itga6/Itga7, Itgb1*) plus *Cd40*

Transcriptional analysis revealed active communication between ECs and PCs. We identified several mechanisms of communication, including gap junctions, adhesive interactions, and paracrine signaling (Fig. 3a, 3d). The size and color of the dots in the figures indicated interaction probability and relative expression in the sender and receiver populations, respectively, highlighting bidirectional signaling between ECs and PCs in the SV (Fig. 3a). Specifically, gap junctions were observed with connexin pairs GJA1 (CX43) and GJC1 (CX45). At adhesion sites, endothelial integrin pairs and dystroglycan (DAG1) interacted with basement membrane ligands, consistent with the presence of PC-enriched transcripts for laminin and type IV collagen. We also noted pro-angiogenic signals from PCs to ECs, including PGF-FLT1 and MDK-NCL, as well as instructive signals from ECs to PCs, including PDGFB/PDGFA-PDGFRB, DLL4/JAG1/2-NOTCH2/3, GAS6-AXL, and EDN1-EDNRA. In contrast, the brain EC-PC signaling map was simpler and more EC-instructive (Fig. 3b) [9]. This pattern suggested a less transcytotic, more barrier-like endothelium that relied on EC-driven mural instruction and chemokine/adhesion tuning rather than PC-delivered matrix guidance.

At the ultrastructural level, we frequently observed numerous vesicles in both ECs and PCs (Fig. 3c). Further analysis indicated that vascular cells displayed vesicle-trafficking characteristics (Fig. 3d). PCs expressed *Cav1/2*, along with components of the clathrin-mediated endocytosis machinery (*Cltc/Ap2m1/Dnm2/Epn2*) and small extracellular vesicle markers (*Cd63/Cd81/Cd9*). ECs co-expressed *Cav1/2* with *Cavin1/2* and *Plvap*, along with clathrin genes and small EV markers (*Cd81/Cd9*), with *Cd81* relatively more abundant in PCs and *Cd9* more prevalent in ECs (Fig. 3d). Together, these data indicated intrinsic trafficking signatures and a greater number and diversity of EC-PC signals in a more angiogenic and remodeling-competent niche compared with brain EC-PC communication.

### 3D in vitro angiogenesis assays reveal dynamic interaction between ECs and PCs during sprouting angiogenesis

**Figure 4:**
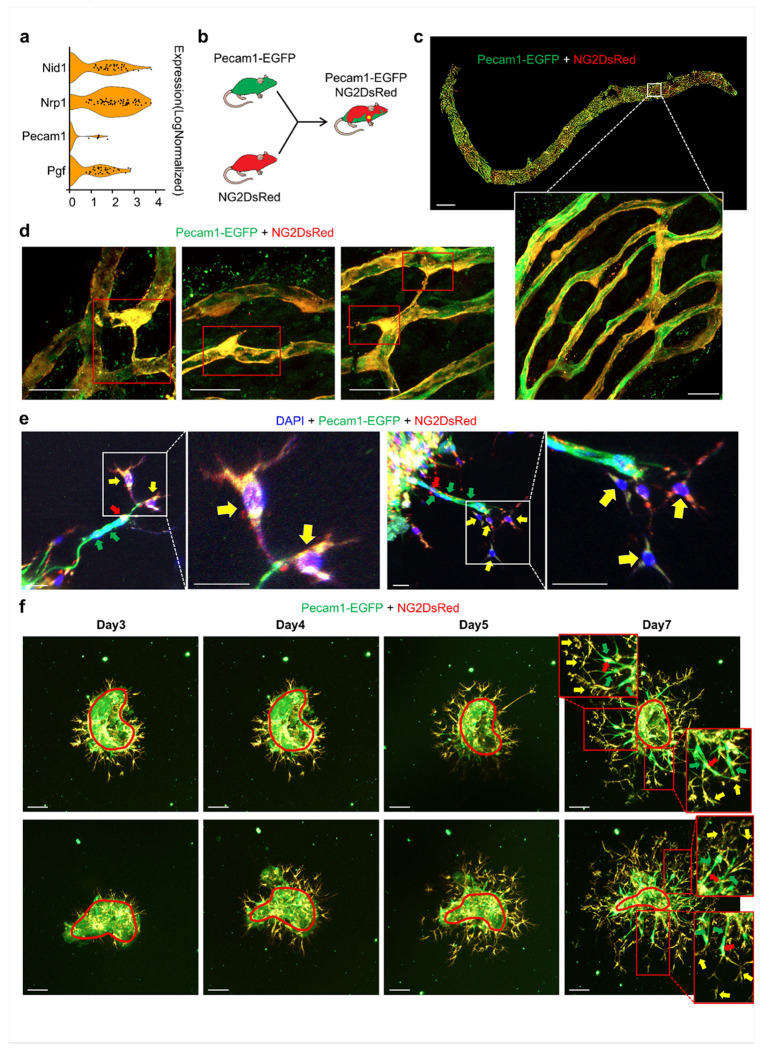
Tip-cell markers in PCs and NG2+/PECAM-1+ tip behavior in explant culture. (a) Expression of tip-cell markers and Pecam1 within the PC population by scRNA-seq (b) The creation of the dural ECPC reporter mouse model by crossing an Pecam1-EGFP mouse with an NG2DsRed mouse (c) Whole-mount SV showing ECs (green), PCs (red), and dual-positive NG2+/PECAM-1+ cells (yellow); Scale bar: 100 μm; The boxed region is shown magnified below;Scale bar: 20 μm(d) Magnified regions of whole-mount SV highlighting migrating NG2+ PCs co-expressing PECAM-1;Scale bar: 20 μm (e) SV explants cultured in growth-factor reduced Matrigel for 7 days and High-magnification images of tip cells at the sprout front co-expressing NG2 and PECAM-1;Scale bar: 20 μm (f) Slower-cadence live imaging of SV explants cultured in normal Matrigel from day 3 to day 7; Tissue bodies are circled; Scale bar: 100 μm; Boxed regions are shown at higher magnification; yellow arrows indicate tip cells, green arrows indicate EC stalk cells, and red arrows indicate recruited PCs

Previous research demonstrated that PCs function as tip cells, actively promoting the formation of new vascular sprouts [8]. However, the detailed mechanisms underlying this process remained incompletely understood. We first asked whether strial PCs carried tip-associated transcripts: scRNA-seq showed tip-cell marker expression within the PC population together with Pecam1 signal (Fig. 4a). To visualize tip dynamics in tissue, we used a dual reporter cross (Pecam1-EGFP × NG2-DsRed) that labeled ECs in green and PCs in red (Fig. 4b). In whole-mount SV, we observed NG2+ PCs (red), ECs (green), and cells with overlapping NG2/PECAM-1 signal (yellow) (Fig. 4c). Higher levels of PECAM-1 signal were especially observed in PCs that exhibited migratory characteristics such as protrusions or extended filopodia and detachment from blood vessel walls, features associated with normal angiogenesis or pathology after noise trauma [22] (Fig. 4d). In 3D strial explants, we noticed that PCs occupied the sprout front and exhibited tip-like behavior, initiating vascular sprouting. Specifically, during sprouting angiogenesis, we observed NG2+ tip PCs migrating forward, extending filopodia, and co-expressing PECAM-1 as leading cells. These cells drove the formation of a PECAM-1+ endothelial tube behind the specialized PCs. Meanwhile, NG2+ PCs with relatively low levels of PECAM-1 wrapped around the developing green fluorescent–labeled endothelial tube, providing stability to these emerging vascular structures (Fig. 4e). In slower-cadence live imaging, tip cells emerging from the tissue margin were consistently dual-positive (NG2+/PECAM-1+) over subsequent days, supporting PC-front tip presence with PECAM-1 detectability at the front (Fig. 4f). By contrast, in the brain, we did not detect NG2/PECAM-1 overlaps in whole-mount tissue (Fig. S5), consistent with lower angiogenic activity in brain microvessels. Together, these data supported PC-front, dual-labeled tips in the cochlea rather than non-overlapping, non-sprouting brain vasculature, highlighting tissue-specific angiogenic competence in the SV. Our findings positioned PCs as a driving force behind vascular sprouting in angiogenesis.

### PC progenitors persist in the stria vascularis and contribute to both PC maturation and angiogenic sprouting

**Figure 5:**
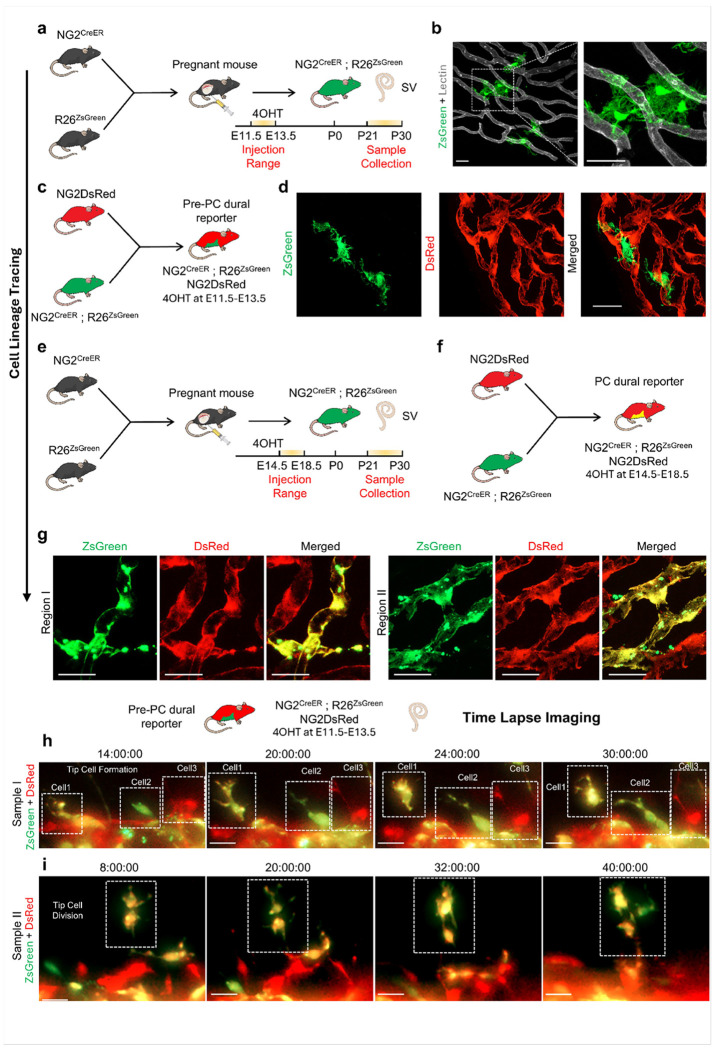
Fate mapping and live imaging identify NG2-lineage PC progenitors in the stria vascularis. (a) Experimental setup of 4OHT injections between E11.5 and E13.5 to label PC progenitors (b) Representative image of PC progenitors (green) in NG2-CreER-ZsGreen mouse stria labeled with lectin (c) The creation of the pre-PC dural NG2-CreER-ZsGreen; NG2DsRed transgenic mouse model by crossing an inducible NG2-CreER-ZsGreen mouse with an NG2DsRed mouse, with Cre recombination occurring at E11.5-E13.5 (d) Representative image of PC progenitors (green) in dural NG2-CreER-ZsGreen; NG2DsRed mouse stria (e) Experimental setup of 4OHT injections between E14.5 and E18.5 to trace progenitor maturation (f) The creation of the PC dural NG2-CreER-ZsGreen; NG2DsRed transgenic mouse model by crossing an inducible NG2-CreER-ZsGreen mouse with an NG2DsRed mouse, with Cre recombination occurring at E14.5-E18.5. (**g**) Representative image showing PC progenitors (green) transferring PCs in dural NG2-CreER-ZsGreen; NG2DsRed mouse stria (h) Time-lapse imaging of strial explant (sample I) from a dural NG2-CreER-ZsGreen; NG2DsRed mouse triggered at E13.5 at multiple time points showing three distinct cell types: one that co-expresses both reporters (cell 1), one that initially expresses only NG2-CreER-ZsGreen but gradually turns yellowish (cell 2), and one that only expresses NG2DsRed (cell 3) (i) Similar cellular events were observed in sample II. The highlighted tip cell is in the process of dividing; Scale bar: 20 μm

To determine whether tip-leading PCs arose from resident PCs or from PC progenitors, we established an inducible PC dual fluorescent reporter mouse model, using hydroxy-tamoxifen (4OHT) induction during embryonic periods both before and after PC specification (Fig. 5a–g). Early induction between embryonic days E11.5 and E13.5, prior to PC integration, labeled an NG2-lineage perivascular population detectable in the adult SV (Fig. 5a). These ZsGreen^+^/DsRed^−^ cells were positioned along microvessels and extended processes toward the vessel wall without wrapping around them, while DsRed+ PCs were observed resting on blood vessels (Fig. 5b, 5d). In contrast, later induction between E14.5 and E18.5 labeled NG2-lineage cells that adopted a mature PC configuration exhibiting wrap-around morphology along vessels, consistent with maturation into PC identity (Fig. 5e–g). Lineage tracing revealed that NG2+ cells were PC progenitors.

To further investigate whether NG2-lineage progenitor cells maintained a strong commitment to PCs in adult mice, which play a crucial role in new blood vessel formation, we used *ex vivo* SV explants embedded in normal Matrigel, stimulated by Vegfa165, and performed time-lapse imaging on a Zeiss CellDiscoverer 7 microscope. In this dual-fluorescent background, resident PCs were DsRed+, while prenatal NG2-lineage non-PC progenitors were ZsGreen+ near blood vessels (Fig. 5c–d). Across independent preparations, we observed and tracked the migration and recruitment of prenatal NG2-expressing cells from perivascular niches as new vascular branches formed. We captured three distinct types of tip cells in Sample I: (1) ZsGreen+/DsRed+ dual-positive; (2) initially ZsGreen-only that gradually gained DsRed; and (3) DsRed-only PCs leading the sprout (Fig. 5h). Similar cellular dynamics were recorded in Sample II, including a dividing tip cell (Fig. 5i). These data identified NG2-lineage perivascular progenitors in the adult SV and showed that both resident PCs and PC progenitors could become tip cells leading vascular sprouts in adulthood, establishing dual cellular sources for tip behavior in the mature cochlea.

## Discussion

This study revealed, for the first time, a distinct transcriptional profile for vascular cells in the strial vasculature. Strial ECs are homogeneous and exhibit a high regenerative potential compared with the BBB in the central nervous system. In contrast to ECs, there are two subclasses of PCs present in the ISFBB. PCs and ECs communicate actively, demonstrating a significantly greater number of EC-PC signaling pathways than those in the BBB, most of which are associated with angiogenic process. Additionally, we identified PC progenitors within a vascular niche, where both PCs and their progenitors facilitate angiogenesis by acting as tip cells and transforming into EC-like cells. This research underscores the unique molecular signature of the ISFBB and highlights the potential of PCs for vascular regeneration, particularly in restoring hearing when the cochlear blood barrier is dysfunctional.

### Uniform ECs and diverse PCs in the stria vascularis

The ISFBB comprises ECs lining the inner surface of blood vessels in the SV, protecting the inner ear from toxins while allowing essential ions and nutrients into the cochlea [5]. Despite the significance of strial ECs, little is known about their transcriptional features, partly due to difficulties in isolating high-quality ECs from the smaller cochlear microvasculature. In this study, we used scRNA-seq to analyze freshly isolated ECs and PCs from the adult mouse cochlea. The results revealed a distinct genetic signature and a strong capacity for angiogenesis in strial vascular ECs. Unlike the heterogeneity observed in ECs from other organs [14–16], strial ECs exhibited a homogeneous profile, as canonical markers for arterial, venous, and capillary subtypes as well as tip and stalk state markers did not distinguish between populations (Fig. S2a-b) [17, 18]. Most of the strial vessels are predominantly capillaries [5]. Analysis of whole-cochlea scRNA-seq suggests strial vasculature is predominantly capillary (~ 80%) [23]. The homogeneity observed in our dataset appears to reflect the nature of the strial microvasculature. This may also be due to the limitations of our isolated EC population, which aims for high quality and viability. Strial ECs demonstrated high expression of key endothelial genes, including *Kdr, Flt1, Pecam1*, and *Cdh5*, as well as the energy transporter *Slc2a1 (Glut1)*, corroborating protein-level findings [24]. When comparing ISFBB ECs to brain ECs through DE followed by PANTHER analysis, we found that the genes differentially expressed in strial ECs were enriched for pathways related to angiogenesis, including PDGF signaling (P00047), blood vessel growth (P00005), EGF receptor signaling (P00018), and TGF-β signaling (P00052) (Fig. 1e) [9]. Together, our data indicate that strial ECs form a uniform and interconnected barrier that regulates vascular permeability, facilitates energy transport, and is transcriptionally primed for angiogenic remodeling.

The ISFBB also has a high PC population, which interacts closely with ECs. Our data reveal two transcriptionally distinct subclasses of PCs with complementary localizations and functions. Type 1 PCs (*Tagln*+/*Acta2*+, approximately 10% of PCs) are in pre- and post-capillary zones and have prominent cell bodies with several ring-like processes surrounding vessels. In contrast, Type 2 PCs (*Kcnj8*+/*Abcc9*+) are distributed throughout the capillary network and typically have rounded cell bodies and a few vessel-parallel processes that extend along endothelial tubes (Fig. 2a–f). GO Biological Process analysis of the top 500 genes shows that Type 1 PCs are enriched in laminar shear response, muscle contraction, Notch signaling, and collagen biosynthesis regulation. These features suggest that Type 1 PCs play roles in blood-flow regulation, vasomotor support, and extracellular matrix (ECM) modulation at capillary border zones (Fig. 2g). AUROC-ranked top genes further support this contractile/matrix role—such as upregulation of *Vim*, which enhances PC adhesion and motility while increasing *Tagln, Dstn*, an actin-depolymerizing factor, which contributes to smooth muscle programs; and *Mustn1*, a microprotein secreted by arteriole-associated smooth muscle cells that promotes ECM remodeling—together supporting a force-coupling function (Fig. S3e) [25–27]. In contrast, Type 2 PCs are strongly enriched for basement-membrane organization, vascular transport, tissue homeostasis, regulation of VEGFR signaling, cell-matrix adhesion, integrin-mediated signaling, blood circulation, and positive regulation of intracellular signal transduction, indicating a matrix-interactive, signaling-competent capillary PC state that supports barrier/transport functions and tunes endothelial responsiveness (Fig. 2h). Consistently, AUROC-top markers link Type 2 PCs to ECM-adhesion signaling, membrane/regulatory signaling, and ion/redox handling: *Vtn/Sparc* provide ECM-based routes for PC–EC coupling and barrier control [28, 29], *Malat1* points to stress-adaptive/transcriptional regulation [30], and *Cp* suggests local iron buffering (Fig. S3e) [31]. Given their K-ATP composition and the presence of nearby *Kcnj10*+ intermediate cells, Type 2 PCs likely sense metabolic and potassium micro-gradients. Together with ion currents derived from the epithelium, they may contribute to endolymph homeostasis and support the endocochlear potential [7, 19, 32]. Compared with brain pericytes, cochlear PCs show a relatively stronger angiogenesis-linked transcriptional bias. In our cochlea-versus-brain differential analysis, cochlear PCs preferentially upregulated pathways associated with sprouting and matrix interaction—including Notch, integrin, endothelin, and TGF-β signaling—consistent with a remodeling-competent state in the stria vascularis.

### Active and angiogenic EC-PC communication and PC-led tip behavior in the adult stria vascularis

Interactions between PCs and ECs are essential for maintaining the integrity and functionality of blood vessels. In this study, we identified that EC-PC interactions occur through direct contact, matrix anchorage, and vesicle-mediated signals in a coordinated manner. Several mechanisms of EC-PC communication were highlighted, including gap junctions (GJA1/CX43, GJC1/CX45), tight junction-associated adhesion (JAM2/JAM3), adhesive cell-matrix interactions, and paracrine signaling (Fig. 3a, 3d). The adhesive interactions are anchored by basement-membrane components such as collagen IV and laminins, which engage specific endothelial integrin pairs (ITGA1/ITGB1, ITGA3/ITGB1, ITGA6/ITGB1) and DAG1 (Fig. 3a). This is consistent with the presence of type IV collagen and laminin-enriched PCs in the cochlear lateral wall. Additionally, inner-ear literature indicates that collagen IV, laminin, and heparan sulfate proteoglycans (HSPGs) regulate labyrinthine fluid dynamics and cochlear barrier function [33]. PCs are recognized producers of basement-membrane components (type IV collagen, laminins, nidogen-1, perlecan, fibronectin) and modulators of matrix metalloproteinases (e.g., MMP2/MMP9), thereby stabilizing the vascular wall and tuning EC behavior [34–37]. In line with this, we observed strong communication probabilities between COL4A1-COL4A6 and integrin receptors, emphasizing collagen IV-integrin coupling as a primary adhesive mechanism. Pathologically, both animal models of Alport syndrome and human patients demonstrate compromised integrity of the matrix interface [38, 39]. Individuals with Alport syndrome often experience progressive hearing loss due to mutations in *Col4a3, Col4a4*, and *Col4a5*, underscoring the critical role of basement-membrane integrity in auditory outcomes [39]. Additionally, defects in basement-membrane collagen can be primary causes of hearing loss—for instance, COL4A2-related basement-membrane dysfunction has been linked to syndromic hearing loss via capillary and microcirculatory disturbance in the cochlea [40]. Beyond adhesion, strial vascular cells display distinct characteristics in vesicle trafficking: PCs express *Cav1/2* with *Cltc/Ap2m1/Dnm2/Epn2* and small-EV markers (*Cd63/Cd81/Cd9*), whereas ECs co-express *Cav1/2* with *Cavin1/2* and *Plvap*, along with clathrin genes and small EV markers (*Cd81/Cd9*) (Fig. 3d). Notably, *Cd81* is relatively more abundant in PCs, whereas *Cd9* is more prevalent in ECs. At the ultrastructural level, we observed numerous vesicles in both ECs and PCs; their precise identities (e.g., endosomes) remain to be defined (Fig. 3c). In contrast, the relationship between brain ECs and PCs is relatively simpler and more EC-instructive (Fig. 3b) [9]. This correlates with prior reports that brain endothelium is less transcytotic and more barrier-like, relying on EC-driven mural instruction and chemokine/adhesion tuning rather than PC-delivered matrix guidance [41–43]. Overall, our transcriptomic analysis of vascular-cell interactions reveals a complex communication architecture in the SV: PCs supply and remodel the basement membrane (COL4A1-COL4A6/laminins) and deliver vesicular signals, while ECs integrate these matrix and paracrine inputs via integrins/DAG1 and signaling receptors to coordinate barrier function.

In addition to the communicative signals mentioned above, we also identified a directional, angiogenesis-skewed signaling architecture between PCs and ECs in the adult SV. On the PC-EC axis, PGF-FLT1 (VEGFR1) and MDK-NCL emerged as prominent pro-angiogenic cues, consistent with perivascular cells priming EC migratory/survival programs. On the EC-PC axis, we detected instructive signals that traditionally support mural recruitment, guidance, and specialization, including PDGFB/PDGFA-PDGFRB, DLL4/JAG1/2-NOTCH2/3, GAS6-AXL, and Edn1-Ednra. Compared with the brain, the cochlea exhibited a richer, more diverse repertoire of PC-EC pathways, with a larger proportion linked to angiogenic and matrix-interaction processes (Fig. 3a, 3b). We observed that several genes associated with tip cells, particularly *Nrp1*, which encode neuropilin-1 (a VEGF coreceptor), were expressed by PCs (Fig. 4a). Prior studies demonstrated that PCs could function as tip cells that lead angiogenic sprouts, challenging the traditional view that only specialized ECs possess tip-cell identity. The presence of tip-associated transcripts within the PC compartment supported the hypothesis that PCs may lead the angiogenic sprouting. To further differentiate between ECs and PCs as potential tip sources in the SV, we used cell-type-resolved reporters with a functional 3D sprouting assay and slower-cadence live imaging under VEGFA stimulation, allowing direct, simultaneous observation of DsRed-labeled PCs and EGFP-labeled ECs (Fig. 4b). In whole-mount SV, we observed NG2+ PCs (red), ECs (green), and a subset of dual-positive NG2+/PECAM-1+ cells (yellow) (Fig. 4c–d). In explants, the invasive front was occupied by NG2+ tip PCs that migrated with filopodia and co-expressed PECAM-1, while NG2+ PCs without PECAM-1 sometimes wrapped the trailing PECAM-1+ endothelial tube (Fig. 4e). These observations aligned with our prior report that PCs acting as tip-leading cells initiate sprouting in culture. These findings also indicated that tip PCs were specialized PCs, distinct from PCs wrapping newly generated branches. By contrast, brain whole-mounts and explants lacked NG2/PECAM-1 overlap (Fig. S5). We assessed the activity of angiogenesis in the brain under the same conditions, but we did not see significant angiogenesis during the 5-day culture period (data not shown). However, the lower level of angiogenic activity in the adult brain compared to the adult cochlea does not imply that angiogenesis does not occur in the brain. In fact, significant angiogenesis activity has been consistently observed when the brain is under hypoxic conditions or during the development of brain tumors[44]. Finally, we identified both PC subclasses, contractile Type 1 and less-contractile Type 2, as participating in sprouting as tip cells (Fig. S4), suggesting that angiogenic capacity is shared across the PC lineage rather than confined to a specific subtype.

### The developmental origin of PCs and the source of tip cells in the adult stria vascularis

Are tip cells derived from existing PCs or from PC progenitors? PCs are generally believed to originate from embryonic mesenchyme and the ectoderm-derived neural crest [10, 11]. However, studies have shown they can also be trans-differentiated from tissue-resident mesenchymal stem cells [45] and glial populations [46, 47]. We previously reported a significant presence of NG2 (*Cspg4*)-expressing cells in the SV of the adult cochlea that express NG2 during early development but lose or fail to maintain NG2 expression in adulthood [48]. The presence of prenatal NG2-expressing cells in perivascular niches raised the question of whether these cells are PC precursors. In this study, we used lineage tracing and fate mapping with an inducible dual-fluorescence NG2 reporter model (NG2-CreER-ZsGreen; NG2-DsRed). We activated Cre recombinase between E11.5 and E13.5, prior to the emergence of PCs, and observed ZsGreen+ non-PC cells in the perivascular niche (Fig. 5a–d). When Cre was activated after E14.5 (E14.5-E18.5), some NG2-ZsGreen+ cells differentiated into PCs that integrated into blood vessels, indicating that NG2-lineage cells can serve as PC progenitors (Fig. 5e–g). To further investigate the origin of tip cells during new branch assembly *in vitro*, we obtained tissue samples from an animal model before PC appearance and used automated time-lapse imaging to track the spatial dynamics of all NG2-expressing cells (Fig. 5h–i). Our findings indicated that both PC progenitors and existing PCs could function as tip cells involved in angiogenesis.

### Summary

This study introduces a unique molecular signature for the cochlear vasculature within the SV, emphasizing the transcriptional uniformity of ECs and the diversity of PCs. It also highlights the interactions between PCs, their progenitors, and ECs that drive angiogenesis and demonstrate regenerative capabilities. Dysfunction of this blood barrier has been linked to hearing disorders, suggesting that targeting PCs and their signaling pathways may offer a promising approach for promoting vascular regeneration and restoring hearing.

## Methods

### Animal models

The mouse strains used in this study include B6.Cg-*Gt(ROSA)26Sor^tm6(CAG-ZsGreen1)Hze^*/J (R26^ZsGreen^, JAX # 007906), Tg(Cspg4-DsRed.T1)1Akik/J (NG2^DsRed^, JAX #008241), B6.Cg-Tg(Tagln-cre)1Her/J (Tagln^Cre^, JAX #017491), B6. C57BL/6-Tg(Pecam1-EGFP)1Linli/J (Pecam1^EGFP^, JAX # 033111), Tg(TIE2GFP)287Sato/J (TIE2-GFP, JAX #003658), and B6. Cg-Tg(Cspg4-cre/Esr1*)BAkik/J (NG2^CreER^, JAX # 008538), which were originally purchased from Jackson Laboratory. All transgenic mice were inbred in the lab and were validated and genotyped for the study. Both male and female mice were used.

For fate mapping experiments investigating PC progenitors, R26^ZsGreen^ mice were crossed with NG2^CreER^ mice. Cre-mediated recombination was induced between embryonic days 11.5 and 13.5 or 14.5 and 18.5. The embryonic development stage was defined relative to the detection of a vaginal plug, designated as E0.5. To induce recombination, pregnant dams were administered intraperitoneal injections of 4-hydroxytamoxifen (4-OHT, Hello Bio, Cat# HB6040) at a dose of 75 μg/g body weight once between the injection windows, supplemented with 37.5 μg/g of corn oil (Sigma Aldrich, Cat# C8267) to mitigate the risk of fetal abortion. All animal experiments reported were approved by the Oregon Health & Science University Institutional Animal Care and Use Committee (IACUC IP00000968).

### Stria vascularis dissections and immunostaining

The cochleae were isolated and fixed in 4% paraformaldehyde (PFA) overnight at 4°C. Following fixation, tissues were washed three times with 1× PBS and the strias were carefully dissected. For TAGLN (Abcam, Cat# ab14106), VE-Cadherin (Abcam, Cat# ab33168), CD31 (Abcam, Cat# ab7388), and GLUT1 (Abcam, Cat# ab15309) labeling, strias were first permeabilized in 0.5% Triton X-100 for 30 minutes at room temperature (RT), followed by blocking in 10% GS and 1% bovine serum albumin (BSA) in 1× PBS for 1 hour. Samples were then incubated overnight at 4°C with the corresponding primary antibody, diluted in 1% BSA. For KCNJ8 (Invitrogen, Cat# MA5-27679) labeling, samples were incubated in a blocking/permeabilization solution consisting of 0.25% Triton X-100 and 10% goat serum (GS) in 1× PBS for 1 hour at RT. Tissues were then transferred into the primary antibody solution, diluted in the same blocking/permeabilization buffer, and incubated overnight at 4°C.

After primary antibody incubation, all samples were washed three times with 1× PBS and then incubated with fluorescence-conjugated secondary antibodies (diluted in blocking/permeabilization buffer) for 1 hour at RT. Samples were again washed three times with PBS, mounted on glass slides using Antifade Mounting Medium with DAPI (Vector Laboratories, Cat# H-1000), and imaged under a 10x, 20x, or 63x objective using either an Olympus FV1000 laser-scanning confocal microscope (Olympus, Japan) or Zeiss LSM980 confocal microscope (Zeiss, Germany). Specifically, we utilized Z-stack (1 μm per step, ~ 20 steps) imaging to capture the desired layer of the SV. Laser intensity, step size, and resolution were kept consistent across all scans to ensure comparability.

### Cochlea frozen sections and immunolabeling

Cochleae from the WT young mice were dissected and fixed in 4% PFA overnight. Following fixation, tissues were decalcified overnight using bone decalcifier (Decal Chemical Corporation, Tallman, NY), dehydrated in 30% sucrose, and embedded in OCT compound (Sakura Finetek, Cat# 62550). Serial mid-modiolar cryosections (7 μm) were prepared and washed in PBS for 15 min to remove residual OCT. Sections were permeabilized with 0.5% Triton X-100 (Sigma-Aldrich) for 30 min and blocked for 1 h in 10% normal goat serum prepared in PBS. Primary antibody incubation was performed overnight (12–16 h) at 4°C using anti-GLUT1 (Abcam, Cat# ab15309). After washing, sections were incubated with fluorescence-conjugated secondary antibodies for 1 hour at 37°C. Finally, samples were washed for 30 min, mounted using Antifade Mounting Medium with DAPI (Vector Laboratories, Cat# H-1000), sealed in 20% glycerol, and imaged with an FV1000 Olympus laser-scanning confocal microscope (Olympus, Japan).

### Transmission Electron Microscopy

Cochleae from 6-week-old C57/6J mice were perfused and fixed in a fixative of 4% (wt/vol) PFA and 0.1% (vol/vol) glutaraldehyde (Electron Microscopy Sciences, Hatfield, PA) in 0.1 mol/L phosphate buffer overnight. Strial tissues were dissected and post fixed in 1% osmium (Electron Microscopy Sciences, Hatfield, PA). Tissues were dehydrated with a graded alcohol series and embedded in Embed 812 (Electron Microscopy Sciences). Sections (1 μm thick) were made to identify the blood vessels with light microscopy, when the area of interest was visible. Ultrathin sections (80 nm thick) were obtained, using a diamond knife (Diatome, Hatfield, PA) and an AO/Reichter ultracut-E microtome (Microtome Service Company, Liverpool, NY), mounted on formvar-coated single-slot copper grids, and counterstained with 1% aqueous uranyl acetate (Electron Microscopy Sciences) for 1 hour. Systematic analysis was made in tissue sections containing the microdissected stria vascularis. Transmission electron microscopy observations and digital image capture were made using an FEI Tecnai transmission electron microscope T12 TEM-120 KV (Hillsboro, OR). All sections are systematically analyzed at low (1100× to 3200×) and higher-magnification view (4000× to 11,000×). All sections were studied for the presence of vesicles in the ECs, PCs, and perivascular basement membrane alterations (ie, thickening and disruption).

### Ex vivo strial tissue explants and angiogenesis assays

The stria vascularis was microdissected from the cochlea and trimmed into small explants approximately 2–3 mm^3^ in size. Tissues were embedded in Corning^®^ Matrigel^®^ Basement Membrane Matrix (LDEV-free, Cat. #354234) or growth factor reduced (GFR) Matrigel^®^ (LDEV-free, Cat. #354230) pre-coated culture dishes and maintained in a humidified incubator at 37°C with 5% CO_2_. For angiogenesis induction, explants were cultured in phenol red–free endothelial basal medium (ScienCell Cat. #1001-prf) supplemented with recombinant rat VEGFA165 (BPS Bioscience Cat. #91008), penicillin-streptomycin (P/S), and fetal bovine serum (FBS).

For explants embedded in GFR Matrigel, the culture medium consisted of ECM basal medium supplemented with 2% FBS, 1% P/S, and VEGFA165 (50 ng/mL). Sprouting consistently initiated around day 5 and explants were maintained for 7 days. For explants embedded in regular (non-GFR) Matrigel, the culture medium consisted of ECM basal medium supplemented with 10% FBS, 1% P/S, and VEGFA165 (50 ng/mL). Sprouting consistently initiated around day 3 and explants were maintained for 5 days. Culture medium is replaced every 3 days. Explants were subsequently processed for endpoint analysis or prepared for live time-lapse imaging depending on the experimental objective.

### Slower-cadence live imaging

Strial explants from Pecam1-EGFP; NG2DsRed dual-reporter mice were embedded in standard Matrigel and cultured for 3 days under the angiogenic conditions described above. Explants were imaged on a Zeiss LSM 980 confocal microscope using a 10× objective under live-support conditions (5% CO_2_, 37°C) on days 3, 4, 5, and 7. Specifically, we utilized Z-stack (3 μm per step, ~ 70 steps) imaging to capture the desired layer of the explant. Laser intensity, step size, and resolution were kept consistent across all scans to ensure comparability.

### Time Lapse Imaging

Strial explants from NG2-CreER-ZsGreen; NG2DsRed dual-reporter mice (4OHT induced at E13.5) were embedded in standard Matrigel and cultured for 3 days under the angiogenic conditions described above. Explants were then transferred to a Zeiss Celldiscoverer 7 widefield system equipped with environmental control (37°C, 5% CO_2_). Imaging was performed with a Plan-Apochromat 5×/0.35 objective and 2× Optovar (effective magnification 10×) using 2×2 tile acquisition to cover the entire explant. At each timepoint, green and red fluorescence channels were acquired together with transmitted light (brightfield). Fluorescence illumination used LED modules at 470 nm (green channel) and 567 nm (red channel), each at 25% output; brightfield used the transmitted-light LED at 1% output. Exposure times were 200 ms (470 nm), 250 ms (567 nm), and 5 ms (brightfield). Images were collected on a Hamamatsu camera via a 1× camera adapter. Full Z-stacks were acquired at 3 μm steps, and time-lapse imaging was performed every 2 h for 72 h. Illumination and exposure settings were held constant within each experiment and adjusted only as needed to minimize photobleaching and phototoxicity.

### Single-cell RNA Sequencing Sample Preparation

For single-cell RNA sequencing of stria vascularis (SV) vascular cells, we prepared two independent batches of samples. In the first batch, temporal bones were harvested from Tie2-GFP reporter mice (1–3 months old; n = 10), and in the second batch from NG2-DsRed reporter mice (1–3 months old; n = 10). Immediately after euthanasia, temporal bones were removed and placed in ice-cold Leibovitz’s L-15 medium (Gibco, Cat# 11415064). Under a dissecting microscope, the cochlear lateral wall was opened from apex to base, and the SV was carefully microdissected from each cochlea. For each strain, SV tissue from both ears (20 capsules total per batch) was pooled in 1.5 mL microcentrifuge tubes containing L-15 on ice. Pooled SV tissue from each mouse line was transferred into 1 mL of L-15 supplemented with collagenase IV (Gibco, Cat# 17104019) and DNase I (Roche, Cat# 10104159001) and gently minced with sterile fine scissors. The tissue was incubated at 37°C for 30 min with gentle shaking (~100 rpm) to digest the extracellular matrix. The collagenase solution was then carefully removed and replaced with an EBSS-based (Gibco, Cat# 14170161) papain solution containing papain (20 U/mL) (Wathington Biomedical, Cat# LS003118), 1 mM L-cysteine (Sigma Aldrich, Cat# C7352), 0.5 mM EDTA (Invitrogen, Cat# 15575020), 15 mM HEPES (Gibco, Cat# 15630080), and DNase I. Samples were incubated for an additional 30 min at 37°C. During papain digestion, the tissue was gently triturated every 10 min using a 1000 μL pipette tip to promote formation of a single-cell suspension while minimizing mechanical stress. Following digestion, an equal volume of Leibovitz’s medium containing 20% ovomucoid protease inhibitor (Wathington, Cat# LS003085) was added to quench papain activity. The cell suspension was then passed through a cell strainer (20–70 μm) to remove undigested fragments and large debris. Flow-through cells were collected and centrifuged at 300 × g for 5 min at 4°C. The pellet was washed twice with PBS containing 0.04% BSA, then resuspended in PBS/0.04% BSA for downstream processing.

An aliquot (~10 μL) of each suspension was used to assess cell concentration and viability using an automated cell counter with trypan blue (Gibco, Cat# 15250061). Only suspensions with > 80–85% viable cells were used for single-cell capture. When enrichment of vascular populations was required, single-cell suspensions were subjected to fluorescence-activated cell sorting (FACS) to isolate Tie2-GFP–positive ECs or NG2-DsRed–positive PCs. Gates were set on forward/side scatter to exclude debris and doublets, followed by selection of GFP + or DsRed + events; sorted cells were collected into tubes containing 0.04% BSA in PBS kept on ice. Immediately after counting (and sorting, when applicable), cell suspensions were adjusted to the desired concentration for 10x Genomics capture (typically ~ 500 cells/μL) in PBS/0.04% BSA. Cells from each batch (Tie2-GFP SV and NG2-DsRed SV) were loaded separately onto a 10x Genomics Chromium microfluidic chip, targeting recovery of several thousand single cells per sample. Single-cell encapsulation, cell lysis, barcoded reverse transcription, and library construction were performed according to the manufacturer’s protocol (Chromium Single Cell 3’ chemistry, 10x Genomics). The total amount of time from euthanasia to loading cells onto the Chromium chip was approximately 3 hours for all preparations.

### Single-cell RNA Sequencing Data Analysis

Raw 10x Genomics data were processed in R using Seurat (v5.1.0). Pericyte (PC) and EC (EC) datasets were handled separately for quality control, normalization, and clustering. For PCs, cells were retained if they had 200-5,000 detected genes and 200–20,000 total RNA counts; for ECs, thresholds were 200-6,000 genes and 200 – 30,000 counts. Cells with high mitochondrial content (> 5%) or high ribosomal gene content (> 40%) were excluded. Doublets were identified with scDblFinder and removed prior to analysis. Gene expression was normalized with LogNormalize (scale.factor = 10,000), highly variable features were selected, data were scaled across all genes, and principal component analysis (PCA) was performed. UMAP was computed using the top 10 principal components, and clustering used the Louvain algorithm at resolution 0.5. Cluster identities were assigned by canonical markers (e.g., *Pecam1, Cdh5, Tek, Cspg4, Pdgfrb*), and clusters were manually annotated as EC, Type 1 PC, and Type 2 PC. We identified positive marker genes for each PC subclass using a Wilcoxon rank-sum test (minimum log2 fold change 0.25, expressed in ≥ 10% of cells). From these candidates, we removed housekeeping genes, as well as mitochondrial, ribosomal, and histone genes using name-based filters. The remaining genes were ranked by average log2 fold change within each subclass, and the top 500 per subclass were exported for GO Biological Process enrichment. To infer ligand–receptor signaling between EC and PC populations, we merged EC and PC objects without batch integration to preserve biological differences across reporter lines and analyzed them with CellChat (v2.1.2) using the mouse ligand-receptor database. Putative interactions were filtered by overexpression criteria requiring expression in ≥ 10% of cells, a log fold-change threshold of 0.10, and a nominal p-value filter of 0.20. Communication probabilities were then estimated with a truncated-mean approach (10% trim) on raw log-normalized data, without spatial distance weighting, and scaled by population size, followed by pathway-level inference and network aggregation. The identical pipeline and thresholds were applied to the brain dataset for matched comparisons. Pathway-level and pairwise interactions were summarized with chord and bubble visualizations.

To assess region-specific vascular expression, single-cell RNA-seq for young mouse brain (GSE129788) was processed with the same workflow. Brain cells were filtered at 200-6,000 detected genes, 500-4,000 RNA counts, mitochondrial content < 5%, and ribosomal content < 15%. After normalization, scaling, PCA, UMAP, and clustering, ECs and PCs were extracted by canonical markers and merged separately with their cochlear counterparts. Differentially expressed genes (DEGs) between cochlear and brain ECs and PCs were computed by Wilcoxon rank-sum test with log2 fold change > 0.25 and adjusted p < 0.05. DEG lists were submitted to PANTHER for pathway enrichment. A subset of angiogenesis-annotated genes was highlighted on volcano plots to visualize cochlea-upregulated vascular programs. In parallel, ligand–receptor communication analysis was performed on the brain EC-PC dataset using the identical CellChat pipeline and thresholds as for cochlea, enabling matched, pathway-level comparisons.

## Supplementary Material

This is a list of supplementary files associated with this preprint. Click to download.

• supplementaryfigures.docx

## Data Availability

All data supporting the findings of this study are available within the article and its Supplementary Information. Raw and processed single-cell RNA-seq data generated in this study have been deposited in GEO under accession GSE312224. Public brain single-cell data used for comparative analyses were obtained from GEO accession GSE129788[9].
